# Practices and Attitudes of Bavarian Stakeholders Regarding the Secondary Use of Health Data for Research Purposes During the COVID-19 Pandemic: Qualitative Interview Study

**DOI:** 10.2196/38754

**Published:** 2022-06-27

**Authors:** Stuart McLennan, Sarah Rachut, Johannes Lange, Amelia Fiske, Dirk Heckmann, Alena Buyx

**Affiliations:** 1 Institute of History and Ethics in Medicine TUM School of Medicine Technical University of Munich Munich Germany; 2 TUM Center for Digital Public Services Department Governance, TUM School of Social Sciences and Technology Technical University of Munich Munich Germany

**Keywords:** COVID-19, data sharing, General Data Protection Regulation, GDPR, research exemption, public health, research, digital health, electronic health records

## Abstract

**Background:**

The COVID-19 pandemic is a threat to global health and requires collaborative health research efforts across organizations and countries to address it. Although routinely collected digital health data are a valuable source of information for researchers, benefiting from these data requires accessing and sharing the data. Health care organizations focusing on individual risk minimization threaten to undermine COVID-19 research efforts, and it has been argued that there is an ethical obligation to use the European Union’s General Data Protection Regulation (GDPR) scientific research exemption during the COVID-19 pandemic to support collaborative health research.

**Objective:**

This study aims to explore the practices and attitudes of stakeholders in the German federal state of Bavaria regarding the secondary use of health data for research purposes during the COVID-19 pandemic, with a specific focus on the GDPR scientific research exemption.

**Methods:**

Individual semistructured qualitative interviews were conducted between December 2020 and January 2021 with a purposive sample of 17 stakeholders from 3 different groups in Bavaria: researchers involved in COVID-19 research (n=5, 29%), data protection officers (n=6, 35%), and research ethics committee representatives (n=6, 35%). The transcripts were analyzed using conventional content analysis.

**Results:**

Participants identified systemic challenges in conducting collaborative secondary-use health data research in Bavaria; secondary health data research generally only happens when patient consent has been obtained, or the data have been fully anonymized. The GDPR research exemption has not played a significant role during the pandemic and is currently seldom and restrictively used. Participants identified 3 key groups of barriers that led to difficulties: the wider ecosystem at many Bavarian health care organizations, legal uncertainty that leads to risk-adverse approaches, and ethical positions that patient consent ought to be obtained whenever possible to respect patient autonomy. To improve health data research in Bavaria and across Germany, participants wanted greater legal certainty regarding the use of pseudonymized data for research purposes without the patient’s consent.

**Conclusions:**

The current balance between enabling the positive goals of health data research and avoiding associated data protection risks is heavily skewed toward avoiding risks; so much so that it makes reaching the goals of health data research extremely difficult. This is important, as it is widely recognized that there is an ethical imperative to use health data to improve care. The current approach also creates a problematic conflict with the ambitions of Germany, and the federal state of Bavaria, to be a leader in artificial intelligence. A recent development in the field of German public administration known as *norm screening* (*Normenscreening*) could potentially provide a systematic approach to minimize legal barriers. This approach would likely be beneficial to other countries.

## Introduction

### Background

The COVID-19 pandemic is a threat to global health and requires collaborative health research efforts across organizations and countries to address it. However, lack of integrated, comprehensive, and accessible patient-level data has been identified as a key barrier to COVID-19 research across the globe [1].

A valuable source of information for researchers is the large amount of digital health data collected by health care organizations through electronic health records. Indeed, health care systems worldwide are increasingly using this routinely collected digital health data for biomedical research, enabling large-scale and multidimensional aggregation and analysis of heterogeneous data sources [2]. The increase of such digital data has also created significant opportunities for artificial intelligence (AI) in health care [3]. With the ability to learn from large sets of clinical data, health care AI applications have the potential to support a wide range of activities [4-11], and public and private sector investment in the field continues to grow [12-14]. If data-intensive medicine is able to realize the continuous improvement of health care quality and thereby reduce patient harm, increase health, empower patient decision-making, and improve equity, it would fulfill the core ethical principles of health care [15,16].

However, benefiting from digital health data requires the ability to access and share the data. Single-center databases are also somewhat limited and sharing data across institutions and countries has various potential advantages, including allowing cross-validation of models across institutions to determine which findings are institution specific and which are generalizable and for knowledge discovery to be accelerated [17]. Efforts to create and link databases for secondary-use research, however, can be undermined by concerns about data protection; concerns that are only likely to intensify available data for research become higher resolution and more diverse (eg, medical images and physiological waveforms) [18].

Patients have legitimate interests in controlling access to and use of their health data, and their consent is often required for the use of their personal data if it was not collected for specific research purposes [18]. However, requiring consent for pseudonymized data to be used in secondary-use research cannot only lead to significant administrative and financial hurdles that delay or even impede important research but can also create major selection biases that undermine data representativeness [19]. Although fully anonymized data typically fall outside data protection laws around the world and can thus be freely used and shared, full anonymization is increasingly difficult to achieve given the use of models that can correctly reidentify people in anonymized data sets [20]. Furthermore, irreversible anonymization involves removing essential information required for most large collaborative research projects [21].

The European Union's (EU) General Data Protection Regulation (GDPR) is a key legal framework for the use and exchange of European digital health data for research purposes [18]. The GDPR entered into force in May 2016 but was only applied from May 25, 2018. Although early drafts of the GDPR raised concerns that the regulation may severely restrict data research [22], the final text adopted a more research-friendly approach, and it was thought that the GDPR would have little negative impact on data research overall [23]. However, concerns remain that the GDPR has made many organizations very risk-averse in terms of data sharing, even if the regulation permits such sharing; for example, via scientific research exemption [1]. When health care organizations are overly concerned with individual risk minimization, it threatens to undermine COVID-19 research efforts. It has been argued that there is an ethical obligation to use the GDPR scientific research exemption, particularly during a crisis such as the COVID-19 pandemic, to support collaborative health research [1].

However, the integration of clinical care and clinical research as part of a learning health care system can often conflict with the current regulatory system and raise a number of important ethical, legal, and social implications [24]. The need for more work on determining when patient notification and consent are required has been particularly highlighted, and investigating the views of patients and other stakeholders has been identified as essential to this work [25,26]. Previous international empirical research with patients regarding the secondary use of their data has found widespread support for such activities and willingness to share their data; however, it has also highlighted variations in patient’s wishes regarding notification and consent [27-38]. Previous research with other stakeholders is rather limited. However, a systematic review found that although researchers and health care professionals were generally supportive of data sharing, they raised concerns about access to data, data storage infrastructure, and consent [39]. Research with other stakeholders has highlighted the challenge of balancing the benefits and risks of secondary research [40-42].

Although Germany is known for its strict approach to data protection, it is currently attempting to make health data more useful and meaningful, such as through the Medical Informatics Initiative [43]. During the COVID-19 pandemic, there have also been large research corporations, such as the National University Medicine Research Network. On April 15, 2020, a nationwide standardized template document for patient consent was approved, enabling researchers across Germany to obtain broad consent for the use of pseudonymized health data in accordance with the GDPR. Germany has also taken a number of regulatory efforts to support digital health care. In 2019, it enacted a new Digital Healthcare Act in 2019, which allows digital health applications to be prescribed and reimbursed under statutory health insurance [44,45]. In 2020, the Patient Data Protection Act was passed, and a step-by-step plan to implement electronic health records and complementary applications such as electronic prescriptions was announced [46]. Despite these efforts, however, the successful implementation of such digital health applications has experienced significant delays and is yet to be achieved [47,48]. Nevertheless, Germany, and the federal state of Bavaria in particular, has also set the goal of becoming a leading hot spot and innovation location for AI [49]. Although recent research involving German patients indicates that abolishing consent for secondary research use of clinical data will likely be acceptable to a large majority of patients [28,29], we are not aware of empirical research with other stakeholders such as researchers, data protection officers, and research ethics committee members, regarding their views and use of the GDPR scientific research exemption for secondary-use health data research either during or before the pandemic.

### Objectives

The first German COVID-19 case appeared in Bavaria in January 2020, and Bavaria was one of the most affected states in Germany during the pandemic. In August 2020, the Bavarian State Ministry of Science and the Arts funded the Technical University of Munich’s Faculty of Medicine for COVID-19 research projects. As a part of this program, this project aimed to explore the practices and attitudes of Bavarian stakeholders regarding the secondary use of health data for research purposes in a time of particular need for fast, data-rich research, namely during the COVID-19 pandemic. It was particularly interested in exploring stakeholders’ views and use of the GDPR scientific research exemption for secondary-use health data research, either during or before the pandemic. Such research, even when performed at the local and regional levels to assess attitudes within a specific legal, cultural, and national context, can illuminate and inform the wider challenge of balancing the goals of furthering health research and improving public health with the goal of responsible data use, a challenge that is relevant across the globe.

## Methods

The methods of the study are presented in accordance with the COREQ (Consolidated Criteria for Reporting Qualitative Research) [50].

### Research Team and Reflexivity

#### Personal Characteristics

Interviews were conducted by JL, a male PhD student in sociology. JL, SM, AF, and AB have long-standing experience with qualitative research [24,51-65].

#### Relationship With Participants

No relationship was established between the interviewer and participants before the study, and the participants received limited information about the interviewer. There was no hierarchical relationship between the interviewers and study participants.

### Study Design

#### Theoretical Framework

The theoretical framework used in this study was conventional content analysis [66].

#### Participant Selection

Stakeholders were primarily selected through purposive sampling [67] to ensure that the participants involved in COVID-19 data sharing for scientific research were from different backgrounds. Additional participants were identified using snowball sampling [68]. Participants were contacted by email and provided with information about the study design and aims and rights as participants. Suitable dates for an interview were found for those willing to participate. Verbal consent was obtained from all participants directly before the interview and audio recorded. A total of 17 Bavarian stakeholders agreed to participate in the study and were recruited from 3 groups: researchers involved in COVID-19 research (n=5, 29%), data protection representatives (n=6, 35%), and research ethics committee representatives (n=6, 35%). A total of 6 people who were contacted did not respond to emails.

### Setting

The interviews were conducted between December 2020 and January 2021. All interviews were conducted via a telephone or video call in German. Only the participant and researcher were present during the interview. Overall, 71% (12/17) of the stakeholders were male, and 29% (5/17) were female.

### Data Collection

A researcher-developed semistructured interview guide was developed for each group to guide the discussions (Multimedia Appendix 1). On the basis of the first 2 interviews that did not show any problems, it was decided that no further piloting or adaptation of the interview guides was necessary. No repeat interviews were conducted. Interviews were audio recorded, and no field notes were taken. The interviews lasted an average of 32 minutes (range 20-41 minutes). The interviews were transcribed in full, checked for accuracy, and subsequently pseudonymized. After 17 interviews, a question about data saturation arose, and it was concluded that saturation was reached in the content and attitudes expressed by the participants [69]. The transcripts of the interviews were returned to all participants with an invitation to review the transcription and send any corrections or clarifications; a total of 6 responses were received with minor corrections to syntax.

### Analysis and Findings

Using the interview transcriptions in their original language, JL and SM performed conventional content analysis with the assistance of the qualitative software MAXQDA (version 11; VERBI Software). The analysis commenced after the interviews were completed. Initial themes identified that were common across participants, as well as those unique to individuals, were labeled using a process of open coding. The findings are presented as higher and lower level categories. The other investigators (SR, AF, DH, and AB) reviewed the initial analysis to clarify and refine codes, and conversations among the investigators continued until coding differences were resolved and consensus was achieved. Selected quotes have been translated into English by the researchers using back translation.

### Ethics Approval

This study received a waiver from the Technical University of Munich's Research Ethics Committee.

## Results

### Current Practices

Participants identified systemic challenges in conducting collaborative secondary-use health data research in Bavaria, particularly research that involves sharing health data outside the institution it was collected. These were reported to be preexisting challenges that were independent of the COVID-19 pandemic but were often brought into sharp focus during the pandemic.

Participants described strict handling of patient data in Bavaria, which led to collaborative secondary health data research being conducted primarily only when patient consent (individual or broad) had been obtained, or the data had been fully anonymized. Although patient data could be used within the hospital where it was collected for research or educational purposes without consent, it was reported that sharing pseudonymized data for research purposes outside the hospital where it was collected was generally not possible under Bavarian law without consent. Participants reported that this could make the use of Bavarian patient data in multicenter studies highly bureaucratic and time consuming. Although many participants thought it was important that the GDPR research exemption existed, they reported that it is currently used seldom and very restrictively:

I welcome the fact that this exception exists. Ultimately, it says that the common good takes precedence over individual rights under certain circumstances. That also has to be weighed up. It is also right that ethics committees are called upon to weigh up such things.

P18, ethics committee

And that is the attitude of our ethics committee. We are very restrictive [with the research exemption]. One can ask the patient.

P02, ethics committee

So we have never actually applied this article. And it’s also questionable. So I think that the supervisory authorities will apply very strict standards when it comes to research privilege. So as I said, we have never applied it. It’s really only ever consent that comes into question.

P12, data protection

Participants reported that the strict handling of patient data in Bavaria continued during the COVID-19 pandemic, although COVID-19 project applications were assessed more urgently than other research applications. COVID-19 projects were primarily conducted based on patient consent or anonymization, and participants felt that the GDPR research exemption did not play a significant role during the pandemic. Nevertheless, some research ethics committee representatives reported instances during the pandemic where their committees had allowed the use of pseudonymized data for research purposes without patient consent, as they wanted to allow valuable research to be conducted; however, they saw themselves at risk of breaking the law:

It [the GDPR research exemption] played practically no role for us. It did not have to be forced, because as an ethics committee we made it possible from the outset to work with the data. The patients who were COVID positive and able to give consent were, as far as I know, very willing to agree to this, and those who were not able to give consent because they were too ill, we as an ethics committee stuck our necks out so that their data and samples could also be used.

P10, ethics committee

For many participants, balancing the protection of patient privacy with health research for the common good was at the core of many of these challenges, which was particularly pronounced during the COVID-19 pandemic. Research ethics committee representatives saw it as their responsibility to consider how to best balance these issues:

Of course, there are always situations where two fundamental rights conflict with each other. This is precisely what we have now with this COVID situation [...] Then it is also clearly a matter for society to discuss where we stand. In case of doubt, which of these fundamental rights is more important to us, and in what form, and how can we take the other into account accordingly? And that’s one of the points we have here. I think that’s the case with many ethics committees, that they say, well, the right to data protection, and the right to research, that’s also a right. If there is an extreme contradiction, then we as an ethics committee are authorized, or there is a social consensus that the ethics committees are authorized, to simply look at how strongly the personal right is restricted and how strongly do we restrict the research project. If we had a research project that did not yield any knowledge, then it would not matter. Then the right to privacy always applies. But if we have an emergency situation, then you can probably also say, these retrospective analyses, you might not necessarily need patient consent.

P2, ethics committee

Nevertheless, a number of participants thought that the current strict handling of patient data in Bavaria and Germany generally undermined important health research:

If you ask me personally, I actually find the handling of patient data in Germany too strict. We would need to create legal regulations as other countries have done. Health data protection laws or research data protection laws, for example [...] That would be feasible. But if you follow the discussion about this patient data protection law for [statutory health insurance] patients within the framework of the telematics infrastructure, we Germans, or many Germans, have a fundamental distrust of state institutions and are therefore not prepared to make data available that would really be very helpful for medical research. As a legislator, you probably have to accept that. And in this respect, yes, that is my personal opinion. I think it’s a pity, because we fall behind many other countries in the context of medical research, but yes, that’s a decision of the legislator.

P5, data protection

### Barriers to Collaborative Health Data Research in Bavaria

Participants identified three key groups of barriers that led to difficulties in conducting collaborative health data research in Bavaria: (1) the wider ecosystem at many Bavarian health care organizations, (2) legal uncertainty and risk minimization, and (3) participants’ ethical views.

#### Wider Ecosystem

A number of participants identified issues in the wider ecosystem at many Bavarian health care organizations as underlying barriers to collaborative secondary-use health data research.

#### Medical Informatics

Although participants noted increasing pressure from the German-wide Medical Informatics Initiative to use health data, it was reported that many Bavarian health care organizations were still not using the valuable data they possess. Medical informatics systems were often reported to be inadequate and that there were insufficient people with the right knowledge and skills to implement such systems:

However, this is more likely to come about as a result of pressure from the higher goals of the medical informatics initiative. Simply saying, people, you have to get your data usable at all. And that means there is already an interest among clinics as well. Because they know what a treasure trove of information they have that they don’t even use. Even to the detriment of patients, they don’t use it or can’t use it. Because the information that is available is not used. That is a disadvantage. And I believe that the clinics are already working on this, but there are not enough people to implement it. There is a lack of computer scientists who can implement this, they are being swept off the market because everyone needs one. And data protection experts. So that’s all being built up right now. So I think that’s why it’s a difficult time right now, because I think they’re all being built up on a voluntary basis. There are structures being created. I think medical informatics is a big driver to systematically create structures nationwide, that’s why I say that so often. And then the hospital boards say, yes, finally something uniform. The others are doing the same. Maybe that’s something that helps a little bit.

P20, ethics committee

#### GDPR Implementation

Some participants felt that the basic implementation of the GDPR is still lacking in many Bavarian health care organizations; smaller institutions, in particular, were reported to have insufficient financial and personal resources to adequately implement it. Some participants were also not in favor of using the research exemption until sufficient implementation of the GDPR was achieved:

Well, funnily enough, so after we have been standing here since 2018, three years later, we are still lacking the basic implementation of the GDPR, I think it is the last remaining bastion that is being taken care of here. [Interviewer: So it's sort of, we need to get this place up and running first?] Right. Yes, but actually it's like that. So already data protection per se. So to somehow take all these requirements into account, that's already difficult in itself. And then there's the question of how to implement it, especially technically. And then somehow this research exemption, that would be the crowning glory, so to speak. To be honest, that may be different at other universities that have more money available and are also larger, but not at our university. I think we are basically too small for that and we are not...It's also still a matter of manpower. So you also have to have time and capacity for it somehow. First of all, the basic technical possibilities for complying with the GDPR are lacking at every turn. So I am no longer in favour of this kind of research exemption.

P27, ethics committee

Strict interpretation of the GDPR also made life very challenging at the beginning of the pandemic, with a participant calling for more flexibility and proportion during the pandemic:

The GDPR made life difficult for us in the first few weeks. Because we were partly inhibited in our interaction with the health authorities. That is, so documents that in the Stone Age could only be sent back and forth by fax. Then, when it came to discharging patients to home isolation, etc., we had to do it by fax. That was very tedious. And there, of course, one would like to see a little sense of proportion in the pandemic. And, as the saying goes, the church should be left in the village and not just read the letter of the law. Because sometimes things have to move very quickly in pandemic times. And we really have to adapt requirements to the situational context.

P03, researcher

#### Legal Uncertainty and Risk Minimization

Participants perceived legal uncertainty regarding a number of issues were leading people to be risk adverse in relation to collaborative secondary-use health data research in Bavaria.

#### Bavarian Hospital Act

Participants identified the Bavarian Hospital Act’s Article 27 on Data Protection as a significant barrier. Although participants reported that the act permitted patient data to be used within the hospital for education and research purposes, they repeatedly noted the challenges raised by the requirement in Article 27 that patient data must remain in the custody of the hospital. Participants felt that the provisions of the Bavarian Hospital Act prevented the GDPR research exemption from being used and that patient consent is required if pseudonymized data are shared with third parties for research purposes:

However, this is not based on the research exemption that you allude to in the GDPR. And in my opinion, it is also not possible in Bavaria, because the Bavarian Hospital Act contains special regulations that prevent this.

P05, data protection

That is one point and the other, which I must of course make clear, according to Article 27, Paragraph 4, of the Bavarian Hospital Act, I may indeed conduct research in the hospital with the data as a treating physician, I may commission others, but the data, if I do not have consent, may only leave the house anonymized, which brings us back to the vexed topic of what is anonymised?

P04, data protection

Nevertheless, participants reported that the GDPR research exemption had been incorporated into the Bavarian Data Protection Act but that Article 27 of the Bavarian Hospital Act had not changed:

And in the Bavarian Data Protection Act, such things are partly taken up. [...] In as much, the Bavarian legislator, and it explicitly says above the processing for research purposes, has more or less taken reference to this, has created a regulation, for processing data for research purposes, but has not attached or has not changed Article 27 of the Bavarian Hospital Act.

P05, data protection officer

Participants described how this situation created a great deal of legal uncertainty in Bavaria and led to a general unwillingness to share pseudonymized patient data for secondary-use data research without consent.

#### Vagueness of Law

Regarding the GDPR research exemption, participants reported that as there was significant perceived vagueness in the law in Germany, people avoided using the research exemption to reduce their legal risk:

So they would still need a national law that really allows this. And the laws that currently allow this are very general. So they say that if the research objective cannot be achieved in any other way, then you can also use personal data. [...] The deeper this encroachment is, the less help general clauses and laws are. That’s the case throughout German law. And it’s the same here. So you can’t create a large research project with a deep intrusion into the rights of people and say that I have a general clause that says, if there’s no other way, then that’s how we’ll do it. So as I said, in the Bavarian hospital law it is explicit. It says, yes, you are allowed to do research with the data. And there is also an exception in the data protection law, which gives a suitable guarantee, so to speak. This does not mean that the data may not leave the custody of the hospital. And such, such a clause or such a regulation does not exist nationally, and therefore we have a bit of a problem. So the opening clause GDPR, yes, but national law, very shaky.

P08, data protection

Yes, we have with this [research exemption]. However, I understood the lawyers to say that this exemption is so vague that in case of doubt, if the patient sues, the physician is poorly advised if they do not have the consent. Because we simply have more sensitive data here. So we don’t invoke this research exemption in research or in the ethics committee. In any case, so far, I think there is a consensus among all ethics committees that this [research exemption] is not sufficient.

P02, ethics committee

Of course, we would prefer that or that there would be a concrete definition of how to deal with the research exemption of the GDPR. As far as I know, also from colleagues, we have actually avoided all of this so far and taken the standard route. We see to it that we get consent.

P04, data protection

#### Variations in the Interpretation of the Law

Participants also reported that wide variations in the interpretation of the law could create significant uncertainty and cause researchers to take a very conservative approach to minimize risk. At the local level, participants reported that a lot depended on the makeup of the research ethics committee, whether it was more medically or legally oriented, with a legally oriented ethics committee seen to be more complicated:

The other problem is that ethics committees are structured differently. Whether they are more medically oriented or more legally oriented. So that has to be said honestly. And the more legalistic it becomes, the more complicated it usually is. Because it depends on whether you are looking for a solution or whether you say, I’ll make it easy for myself, I’ll forbid it for now or I’ll put up some kind of hurdle and then I’ll have peace, I’ll close it now. As such it’s very difficult to balance in between them. Because both sides are right, but it’s always a trade-off.

P20, ethics committee

Local ethics committees and data protection officers did not have the same opinion, sometimes resulting in additional hurdles for the researchers:

But even there, there was and is a lot to clarify with data protection officers. Because sometimes there are two hurdles. One is the ethics committees, which are then called upon, and the other is the data protection officers, who do not necessarily always have identical ideas.

P20, ethics committee

Collaborative research at the national and international levels was reported to be generally difficult, with a lack of consensus among data protection commissioners and variations in local laws:

However, in the overarching sense of the exemption regulation, they have to deal with the individual data protection commissioners of the federal states. This makes supra-regional studies extremely difficult because there are so many different opinions of the data protection commissioners and no general opinion can be reached.

P20, ethics committee

Even that doesn’t necessarily help you, because of course data protection is also covered by many area-specific regulations such as the state hospital laws. Yes. This means that what Bavaria now applies does not necessarily apply equally in the case of Rhineland-Palatinate.

P04, data protection

And yet we have 16 or 17 supervisory authorities with different ways of applying the law. Because it is somehow difficult when the data protection commissioner in Baden-Württemberg says something different about the same facts as the data protection commissioner in Berlin. And the further north, the stricter. That has to be said quite clearly.

P05, data protection

One area cited by the participants as resulting in significantly different interpretations was the distinction between pseudonymized data and fully anonymized data:

Is it absolutely anonymised or is it relatively anonymised? Is the recipient of the data anonymous, because he doesn’t know that 150 is Mrs Meier? Or does someone who has passed it on still have a list somewhere that says 150 is Mrs Meier? There are also different interpretations. To be fair, it has to be said that with the strict interpretation it will never be possible to create anonymised data. But here too, as we have learned, there is no agreement among the data protection commissioners, neither in Germany nor throughout Europe.

P20, ethics committee

When sharing data, I have to inform the person responsible, in our case the board of directors, about the legal risk he is taking, because there is no secure interpretation of when pseudonymised data, which cannot be identified by another person, i.e. which are in fact anonymous in the old way of speaking, fall under the GDPR or not. And there are both interpretations in the literature. And research projects that you absolutely want to have, let’s say, then perhaps you tend to say that this is not your own legal basis for dealing with de facto anonymous data. And if at some point the ice becomes too thin, then perhaps in other cases one will say that it is personal data, we cannot do it without consent. [...] I point out every time that there is a certain risk here when you share this data. However, I consider the actual risk of a supervisory authority in Covid times taking action against a research institution that actually exchanges anonymous data to be really small.

P08, data protection

#### Ethical Views

The reluctance to conduct health data research without patient consent also reflected participants’ ethical views. Some participants felt that patient consent ought to be obtained whenever possible to respect patient autonomy, and consequently, that the use of the research exemption should be very limited and as an option of last resort:

So you’re right. I’m a bit reluctant to take this as a license for all kinds of things. Fortunately, it has to be said that many researchers don’t even know this recital. Even within the Ethics Committee, probably not every member knows it in detail either. But yes, I have already said a few times that I would support and welcome something like this. If this is really useful and actually advances science, and I see myself as a scientist in the same way. Then I think, yes, we should use it. But if it serves the laziness somewhere and he says, no, I can do it much more elegantly and it’s all so time-consuming and always inform the poor patient and in the end he doesn’t agree. Then to take refuge in that, I think, is not correct.

P18, ethics committee

Some participants perceived a risk that the research exemption could lead to a *carte blanche* to use patient data and questioned why COVID-19 research should be treated differently from other types of research:

As far as the [research exemption] is concerned, this is viewed somewhat more cautiously, because otherwise it can degenerate into a carte blanche. We do research on humans. I mean, why is Corona now higher-ranking than cancer research or something?

P20, ethics committee

### Facilitators for Collaborative Health Data Research in Bavaria

To improve health data research in Bavaria, participants wanted greater legal certainty regarding the use of pseudonymized data for research purposes without patient consent. In the short term, some participants felt that a clear statement from relevant local authorities clarifying the application of the law would be helpful:

I would suggest the following. Briefly, at least in the short term, a stipulation by the relevant state supervisory authorities that project data and really, I would call it project pseudonymised data, which are not traceable for the recipient of this data under any reasonable conditions and using any normally applicable means or methods, are treated the same as fully anonymised data as far as the transfer of data is concerned and this transfer of data does not require consent. It is equivalent to fully anonymised data as far as data transfer is concerned and this data transfer does not require consent. In the long term, I would like to see an amendment to Article 27 of the Bavarian Hospital Act to the effect that I can say that data may leave the hospital anonymised and correspondingly properly pseudonymised without requiring consent.

P04, data protection

Well, there could be a clear statement by the Bavarian legislator, so to speak, about what data is affected and how, from the point of view of...well, if you could give people more legal certainty and perhaps also a public statement of this information... By making this statement officially, a lot would be gained. If we had something to refer to, whereby, as I said, the Bavarian hospital laws already have this research possibility, but it is always limited in the sharing.

P20, ethics committee

However, most participants ultimately thought that Article 27 of the Bavarian Hospital Act needed to be amended to allow appropriately pseudonymized data to leave the hospital without the explicit consent of the patient:

In my opinion, this can only be done if the legislator amends Article 27, Paragraph 4 of the Bavarian Hospital Act. And not only for Covid data, but I think for research into...So Covid is of course important and currently very high. But there are many other diseases [...] and if there were a legal basis for using this data for research purposes across the board, that would certainly, as I briefly mentioned before, for me personally, yes, that wouldn’t be bad.

P05, data protection

In the long term, I would like to see an amendment to Article 27 of the Bavarian Hospital Act to the effect that data may leave the hospital anonymised and correspondingly properly pseudonymised without requiring consent.

P04, data protection

However, participants also pointed out that this situation cannot be improved by Bavaria alone and saw the need for a federal law for the handling of research data:

To be honest, I don’t see Bavaria as the decisive factor for advancing research in an area like this. You have to talk to more specialised centres, and the German university hospitals are already a good cluster. Although at the level of a university hospital, I would say that a Bavarian regulation would perhaps be easier to implement in parliament, but all the projects of the National University Medicine Initiative alone would not be helped here, because they are all coordinated via Charité.

P08, data protection

I would like to see something like a research law or at least a binding specification of the requirements and a binding harmonisation of the requirements at federal level. We should say that we are enacting a research law and that data should be handled in such and such a way. Taking into account the GDPR, data protection regulations, hospital regulations and other regulations.

P04, data protection

## Discussion

### Principal Findings

This is one of the first qualitative studies examining European stakeholders’ views and use of the GDPR scientific research exemption for secondary-use health data research, either during or before the COVID-19 pandemic. It also aims to add empirical insight to the global debate about the conflicting goals of furthering public health and health research on the one hand and protecting individual privacy and ensuring responsible data use on the other. This study has resulted in two key findings: (1) stakeholders in the German federal state of Bavaria were generally unwilling to use scientific research exemption owing to legal and ethical concerns and (2) stakeholders felt that the current strict handling of patient data is undermining important health research. This study suggests that the balance between enabling the positive goals of health data research and avoiding associated data protection risks can often be heavily skewed toward avoiding risks; thus making it extremely difficult to reach the goals of health data research. This is important as it is widely recognized that there is an ethical imperative to use health data to improve care. The current approach also creates a problematic conflict with Germany’s, and the federal state of Bavaria’s, ambitions to be a leader in AI. However, this is also a challenge for many other countries.

Despite recent research indicating that abolishing consent for secondary research use of clinical data will likely be acceptable to a large majority of German patients [28], Germany and many other countries, including in the EU, are still pursuing a *consent or anonymize* approach. Various authors have argued for several years that this approach undermines data-intensive medicine and that there is a need to specify the appropriate conditions for using a research exemption from consent [21]. Article 9(2)(j) of the GDPR sets out a scientific research exemption for processing sensitive personal data, which could occur without consent if subject to appropriate safeguards and if such rights would render impossible or seriously impair the achievement of the research purpose—see Article 89(1). Such a research exemption has a number of advantages in the context of the secondary use of health data for research purposes. The data from a large number of patients not requiring consent can be covered by the same provision. The existence of the research purpose is also relatively independent of further developments. In contrast, for example, if the data processing is based on Article 9(2)(i) GDPR, which explicitly allows the processing of sensitive personal data if it is “necessary for reasons of public interest in the area of public health,” the processing can only take place as long as it is necessary to protect against the risk. If the situation stops being so dangerous, it would have the consequence that the data could not be used any further, not even to avert more abstract or possible future dangers [70].

However, as this study highlights many countries across the world continue to pursue a restrictive approach regarding the secondary use of patient data and seldom allow the use of data without consent or anonymization. As the participants of this study noted, various regulatory regimes must be considered, both at the national and European levels, with regard to the legal use of data in EU countries. Owing to the primacy of the application of EU law, the lawfulness of data processing is governed by the GDPR. This is supplemented by further general data protection laws at the national level (for Germany, the Federal Data Protection Act), as well as the state level (for Bavaria, the Bavarian Data Protection Act). However, Article 9(2)(i) of the GDPR—in conjunction with Article 89(1)—does not constitute separate authorization for data processing for research purposes [71]. Rather, the member states must make use of this opening clause through their own law. For Germany, this means that because of the federal system and the different competences of the federal government and federal states, the competence is initially based on the national competence regulations. Corresponding regulations can be found in section 27 of the Federal Data Protection Act; Article 25 of the Bavarian Data Protection Act, Act 25, section 27; and in Article 27 of the Bavarian Hospital Act. However, the decisive competence in this area usually lies with the federal states (exceptions may occur in individual cases, but in principle, hospital acts apply to all hospitals in a federal state, regardless of whether they are run publicly or privately or church-run). Furthermore, the regulations of the more specific Bavarian Hospital Act take precedence over those of the general Bavarian Data Protection Act (principle lex specialis derogat legi generali). Article 27(4) of the Bavarian Hospital Act states the following:

1. “Hospital physicians may use patient data insofar as this is necessary within the framework of the hospital medical treatment relationship, for initial, further and continuing training in the hospital, for research purposes in the hospital or in the research interests of the hospital.”
2. “They may instruct other persons in the hospital to do so, insofar as this is necessary for the fulfillment of these tasks; for the purposes of research in accordance with sentence 1, they may permit other persons to use patient data if this is necessary for the implementation of the research project and the patient data remain in the custody of the hospital.”
3. “Such persons shall be bound to secrecy.”
4. “The hospital administration may use patient data to the extent necessary for the administrative processing of patient treatment.”

The regulations are narrower than the general data protection requirements in Article 25 of the Bavarian Data Protection Act. In particular, the Hospital Act requires that the data remain in the custody of the hospital. This restriction is not provided for by the Data Protection Act. However, it is up to the member states to decide in which form and under which further conditions they make use of the opening clause. If the requirements of Article 27(4) of the Bavarian Hospital Act cannot be met, one of the other justification options for data processing from Article 9(2) GDPR must be used. Therefore, the current legal requirements in Bavaria hinder the use of patient data for research purposes. Other German federal states have similar regulations to Bavaria; however, some states (eg, Bremen or North Rhine-Westphalia) have opted for more detailed regulations. There is a need to examine such local regulations in more detail, not only in Germany but also in other countries and how these are affecting the secondary use of patient data.

Recent developments in the field of German public administration known as *norm screening* (*Normenscreening*) could potentially provide a systematic approach to minimize such legal barriers. To encourage digital transformation and reduce existing barriers, some German federal states (eg, Schleswig-Holstein and Saarland) reviewed their entire public state laws to identify all norms that could act as barriers to further digitalization. As a result, the federal state of Saarland identified and categorized more than 1000 rules, and those preventing, or at least complicating digitalization, were removed or at least changed into more moderate forms. Such a screening process could be used to identify all types of legislative obstacles, sort them, and enable fundamental changes. Therefore, it is suggested that a screening process could be used to harmonize and minimize barriers in regulations regarding the use of patient data for research purposes. The focus would be on regulations that are narrower than those required by EU or constitutional law. This screening process would likely be beneficial to all EU members, as member states are required to make use of the GDPR scientific research exemption through their own national law and will therefore likely face similar challenges as those described in Germany. This approach could also, in principle, be applied outside Europe.

In an era of increasing global collaborative health research efforts, however, significant variations in laws regarding this issue are not only a problem within countries but also across countries [18]. Concerns have long been raised that the GDPR allows too much room for interpretation of the regulation by member states on key aspects of data protection, including sufficient methods of pseudonymization, when data are considered fully nonidentifiable, what further limitations should be set on processing sensitive data for research purposes, and sufficient safeguards and conditions for processing data under research exemption [72]. Although this may help recognize local values and norms, it risks undermining the goal of the GDPR to address the heterogeneity of data protection within the EU. The process of norm screening on a national level could potentially help identify already existing similarities between member states as well as detect best practices, which could support the progress on EU-level.

Germany, and Bavaria in particular, prides itself for research impact and innovation potential. However, their current approach to patient data is often heavily skewed toward avoiding risks; thus making it extremely difficult to reach the goals of health data research. This approach also conflicts with their stated ambitions to be leaders in AI. Benefiting from data-intensive medicine, particularly activities driven by AI technologies, requires first and foremost, having access to data. Being very restrictive with secondary patient data use at the same time as pouring significant amounts of public funds into data-intensive medical and medical AI is inconsistent and arguably unethical, as it constitutes a waste of public resources and, at worse, may end up causing patient harm owing to unrepresentative and biased data sets and models [73]. Politicians and policy makers need to take the issue of data access and sharing more seriously.

### Strengths and Limitations

This is a qualitative study that did not collect statistically representative data. However, we included a range of experts who have direct experience with sharing COVID-19–related health data for research purposes in Bavaria, which makes it likely that this study has captured key aspects of a multisided issue. A bias might exist toward the reporting of socially desirable attitudes [74]; however, given that our results are rather critical of current practice, we believe that such a bias is limited. The study was only carried out in Bavaria, and there may be some regional and country-specific differences that might limit the generalizability. Nevertheless, many of the key issues are associated with aspects that are common in other countries (eg, balancing the goals of public benefit of the research with consent and privacy), and these findings are likely to be of wider international interest. The strengths of this study include the fact that it is, to our knowledge, one of the first to investigate stakeholders’ views and use of the GDPR scientific research exemption either during or before the pandemic.

## Appendix

Multimedia Appendix 1 Interview guides.

## Abbreviations

AI: artificial intelligence
COREQ: Consolidated Criteria for Reporting Qualitative Research
EU: European Union
GDPR: General Data Protection Regulation

## Data Availability

Our data include pseudonymized transcripts of interviews, which cannot be made publicly available in their entirety because of (1) the terms of our ethics approval and (2) because participants could be identifiable if the context of the entire transcript. This is in line with the current ethical expectations for qualitative interview research. We provide anonymized quotes within the paper to illustrate our findings (corresponding to transcript excerpts), and the complete interview guide used in the study has been included in the Multimedia Appendix 1.
