# Supplementary material for: Practices and Attitudes of Bavarian Stakeholders Regarding the Secondary Use of Health Data for Research Purposes During the COVID-19 Pandemic: Qualitative Interview Study
Source: J Med Internet Res. 2022 Jun 27;24(6):e38754. doi: 10.2196/38754 (PMC9239567; doi:10.2196/38754)
Supplement: Multimedia Appendix 1 [file jmir_v24i6e38754_app1.docx]

## **Multimedia Appendix 1. Interview Guides**

**Researchers Interview Guide**

[English translation]

1. **How has the pandemic affected your work/research?**

Next, I would like to talk to you about data protection in relation to health data sharing.

1. **Can you tell me about your experience with projects using Covid-19 health data for scientific research?**
2. What is usually the source of these Covid-19 data? (internal, Bavaria, national, EU, international).
3. **What legal justifications are usually used for these Covid-19 projects? (e.g. consent/anonymisation/research exemption) Have there been problems with the use of these legal justifications?**
4. If the GDPR research exemption was used: Do you think the research exemption should be used more often to support Covid-19 research? Does your organisation provide guidelines or technical/organisational measures regarding the research exemption?
5. If the GDPR research exemption was not been used: Are you aware of the GDPR research exemption? If yes: Why have you not used it? Do you think it has a role in supporting collaborative Covid-19 research? Does your organisation provide guidelines or technical/organisational measures regarding the research exemption?

**4. What would you suggest to improve the sharing of Covid-19 health data for scientific research by Bavarian health organisations?**

[German original]

1. **Wie hat die Pandemie Ihre Arbeit/Ihre Forschung beeinflusst?**

Als nächstes würde ich gerne mit Ihnen über den Datenschutz bzgl. Dem Austausch von Gesundheitsdaten sprechen:

1. **Können Sie mir Ihre Erfahrungen mit Projekten mitteilen, bei denen Covid-19 Gesundheitsdaten für die wissenschaftliche Forschung benutzt werden?**
   1. Was ist normalerweise der Ursprung dieser Covid-19-Daten? (intern, Bayern, national, EU, international)
2. **Welche rechtlichen Begründungen werden normalerweise für dieser Covid-19 Projekte benutzt? (z.B. Einwilligung/Anonymisierung/Erlaubnisregelung für Wissenschaft) Gab es Probleme bei der Anwendung dieser rechtlichen Begründungen?**
   1. ***Falls die DSGVO-* *Erlaubnisregelung für Wissenschaft verwendet wurde***: Sind Sie der Meinung, dass die Erlaubnisregelung für Wissenschaft häufiger zur Unterstützung von Covid-19 Forschung verwendet werden sollte? Stellt Ihre Organisation Richtlinien oder technische/organisatorische Maßnahmen bezüglich der Erlaubnisregelung zur Verfügung?
   2. ***Wenn die DSGVO-* *Erlaubnisregelung für Wissenschaft nicht verwendet wurde*:** Ist Ihnen die DSGVO- Erlaubnisregelung für Wissenschaft bekannt? ***Wenn ja***: Warum haben Sie sie nicht angewendet? Glauben Sie, dass sie eine Rolle bei der Unterstützung der gemeinsamen Nutzung von Covid-19 Forschung spielt? Spielen sollte? Stellt Ihre Organisation Richtlinien oder technische/organisatorische Maßnahmen bezüglich der Erlaubnisregelung zur Verfügung?
3. **Was würden Sie vorschlagen, um den Austausch von Covid-19 Gesundheitsdaten für die wissenschaftliche Forschung durch bayerische Gesundheitsorganisationen zu verbessern?**

….

**Data Protection Interview Guide**

[English translation]

1. **Can you tell me about your experience as your organisation's data protection officer with projects using Covid-19 health data for scientific research?**
   1. What is usually the source of these Covid-19 data? (internal, Bavaria, national, EU, international).
2. **What legal justifications are usually used for these Covid-19 projects? (e.g. consent/anonymisation/research exemption) Have there been problems with the use of these legal justifications?**
   1. What are your views on the use of the research exemption provision specifically in the case of Covid-19 research? Should the permission regime be used more often to support Covid-19 research? Do you have any concerns about this?
   2. Do you think that researchers are sufficiently informed about the use of the research exemption? Do you think that university hospitals have implemented sufficient technical/organisational measures regarding the research exemption?
3. **What would you suggest to improve the sharing of Covid-19 health data for scientific research by Bavarian health organisations?**

[German original]

1. **Können Sie mir Ihre Erfahrungen als Datenschutzbeauftragter Ihrer Organisation mit Projekten mitteilen, bei denen Covid-19 Gesundheitsdaten für die wissenschaftliche Forschung benutzt werden?**
   1. Was ist normalerweise der Ursprung dieser Covid-19-Daten? (intern, Bayern, national, EU, international)
2. **Welche rechtlichen Begründungen werden normalerweise für dieser Covid-19 Projekte benutzt? (z.B. Einwilligung/Anonymisierung/Erlaubnisregelung für Wissenschaft) Gab es Probleme bei der Anwendung dieser rechtlichen Begründungen?**
   1. Was sind Ihre Ansichten zu der Nutzung der Erlaubnisregelung für Wissenschaft speziell im Fall der Covid-19 Forschung? Sollte die Erlaubnisregelung häufiger zur Unterstützung von Covid-19 Forschung verwendet werden? Haben Sie diesbezüglich Bedenken?
   2. Sind Sie der Meinung, dass Forscher ausreichend informiert sind über die Anwendung die Erlaubnisregelung? Sind Sie der Meinung, dass die Universitätskliniken ausreichende technische/organisatorische Maßnahmen bezüglich der Erlaubnisregelung umgesetzt haben?
3. **Was würden Sie vorschlagen, um den Austausch von Covid-19 Gesundheitsdaten für die wissenschaftliche Forschung durch bayerische Gesundheitsorganisationen zu verbessern?**

**Ethics Committee Interview Guide**

[English translation]

1. **Can you tell me about your experience in the ethics committee with projects using Covid-19 health data for scientific research?**
   1. What is usually the source of these Covid-19 data? (internal, Bavaria, national, EU, international).
2. **What legal justifications are usually used for these Covid-19 projects? (e.g. consent/anonymisation/research exemption) Have there been problems with the use of these legal justifications?**
   1. What are your views on the use of the research exemption provision specifically in the case of Covid-19 research? Should the research exemption be used more often to support Covid-19 research? Do you have any concerns about this?
   2. Do you think that researchers are sufficiently informed about the use of the research exemption? Do you think that university hospitals have implemented sufficient technical/organisational measures regarding the research exemption?
3. **What would you suggest to improve the exchange of Covid-19 health data for scientific research by Bavarian health organisations?**

[German original]

1. **Können Sie mir Ihre Erfahrungen in der Ethikkommission mit Projekten mitteilen, bei denen Covid-19 Gesundheitsdaten für die wissenschaftliche Forschung benutzt werden?**
   1. Was ist normalerweise der Ursprung dieser Covid-19-Daten? (intern, Bayern, national, EU, international)
2. **Welche rechtlichen Begründungen werden normalerweise für dieser Covid-19 Projekte benutzt? (z.B. Einwilligung/Anonymisierung/Erlaubnisregelung für Wissenschaft) Gab es Probleme bei der Anwendung dieser rechtlichen Begründungen?**
   1. Was sind Ihre Ansichten zu der Nutzung der Erlaubnisregelung für Wissenschaft speziell im Fall der Covid-19 Forschung? Sollte die Erlaubnisregelung häufiger zur Unterstützung von Covid-19 Forschung verwendet werden? Haben Sie diesbezüglich Bedenken?
   2. Sind Sie der Meinung, dass Forscher ausreichend informiert sind über die Anwendung die Erlaubnisregelung? Sind Sie der Meinung, dass die Universitätskliniken ausreichende technische/organisatorische Maßnahmen bezüglich der Erlaubnisregelung umgesetzt haben?
3. **Was würden Sie vorschlagen, um den Austausch von Covid-19 Gesundheitsdaten für die wissenschaftliche Forschung durch bayerische Gesundheitsorganisationen zu verbessern?**
